# The Induction of Cytokine Release in Monocytes by Electronegative Low-Density Lipoprotein (LDL) Is Related to Its Higher Ceramide Content than Native LDL

**DOI:** 10.3390/ijms14022601

**Published:** 2013-01-28

**Authors:** Montserrat Estruch, Jose Luis Sanchez-Quesada, Lorea Beloki, Jordi Ordoñez-Llanos, Sonia Benitez

**Affiliations:** 1 Cardiovascular Biochemistry Group, Biomedical Research Institute Sant Pau (IIB Sant Pau), Barcelona 08041, Spain; E-Mails: jsanchezq@santpau.cat (J.L.S.-Q.); jordonez@santpau.cat (J.O.-L.); 2 Biochemistry and Molecular Biology Department, Autonomous University of Barcelona, Sant Pau Hospital Unity, Barcelona 08041, Spain; E-Mail: lorea.beloki@gmail.com; 3 Biochemistry Department, Sant Pau Hospital, Barcelona 08041, Spain

**Keywords:** electronegative LDL, HDL, cytokines, ceramide, diacylglycerol, lipoprotein aggregation, monocytes, phospholipase C

## Abstract

Electronegative low-density lipoprotein (LDL(−)) is a minor modified LDL subfraction that is present in blood. LDL(−) promotes inflammation and is associated with the development of atherosclerosis. We previously reported that the increase of cytokine release promoted by this lipoprotein subfraction in monocytes is counteracted by high-density lipoprotein (HDL). HDL also inhibits a phospholipase C-like activity (PLC-like) intrinsic to LDL(−). The aim of this work was to assess whether the inhibition of the PLC-like activity by HDL could decrease the content of ceramide (CER) and diacylglycerol (DAG) generated in LDL(−). This knowledge would allow us to establish a relationship between these compounds and the inflammatory activity of LDL(−). LDL(−) incubated at 37 °C for 20 h increased its PLC-like activity and, subsequently, the amount of CER and DAG. We found that incubating LDL(−) with HDL decreased both products in LDL(−). Native LDL was modified by lipolysis with PLC or by incubation with CER-enriched or DAG-enriched liposomes. The increase of CER in native LDL significantly increased cytokine release, whereas the enrichment in DAG did not show these inflammatory properties. These data point to CER, a resultant product of the PLC-like activity, as a major determinant of the inflammatory activity induced by LDL(−) in monocytes.

## 1. Introduction

Modification of LDL is directly related to the initiation and progression of atherosclerosis. Modified LDL can promote subendothelial accumulation of cholesterol and activates the chronic inflammatory response characteristic of atheromatous lesions [1]. Electronegative low-density lipoprotein (LDL(−)) is a minor modified LDL subfraction that is present in circulation [2]. LDL(−) has some potentially atherogenic characteristics compared to native LDL. LDL(−) has a higher content of non-esterified fatty acids (NEFA) and lysophosphatidylcholine (LPC) [3,4], possibly related to its platelet-activating factor acetylhydrolase (PAF-AH) activity, due to the presence of the PAF-AH enzyme [5]. LDL(−) also presents a phospholipase C (PLC)-like activity [6], whose origin is still unknown. It has been suggested that it could be related to other atherogenic traits of LDL(−) [7], such as increased susceptibility to aggregation [8], abnormal apolipoprotein B (apoB) conformation [9] or high binding to proteoglycans [10]. LDL(−) also promotes the release of cytokines, including MCP1, IL6, IL8, IL10 and GRO, in endothelial cells [11], monocytes and lymphocytes [12,13].

In a previous study, we found that HDL counteracted the cytokine release induced by LDL(−) in monocytes [12]. However, the mechanism by which HDL inhibited the inflammatory action of LDL(−) was not clearly defined. This effect could be partly due to the exchange of NEFA between LDL(−) and HDL, since it was previously reported that NEFA are involved in the inflammatory effect of LDL(−). It is also possible that other molecules, generated by the PLC-like activity of LDL(−), could be involved in the inflammatory action of LDL(−) [3,12]. The PLC-like activity in LDL(−) hydrolyzes the polar head of choline-containing phospholipids and preferentially degrades LPC, with medium efficiency sphingomyelin (SM) and with lower efficiency phosphatidylcholine (PC). The products of this hydrolysis are phosphocoline (Pchol), monoacylglycerol (MAG), ceramide (CER) and diacylglycerol (DAG). Pchol is water-soluble and presumably leaves the LDL particle. In contrast, MAG, CER and DAG, which are hydrophobic, would remain retained in the LDL particles [14]. An increased content of MAG, CER and DAG in LDL modifies the surface structure of the particles, leading to their aggregation and fusion by hydrophobic associations [14,15]. PLC-like activity of LDL(−) is mainly present in a subfraction of aggregated LDL(−) [8], suggesting that this activity is involved in LDL aggregation.

Even though LPC is rapidly degraded by the PLC-like activity, MAG would be scarce in LDL, since the amount of LPC is much lower (2%–3% of total phospholipids in LDL) than PC (70%) and SM (20%). CER and DAG are considered as bioactive and inflammatory molecules that promote cell signal transduction. CER participates in the regulation of cell proliferation and differentiation, inflammation and apoptosis in a wide variety of cells, including cells of the immune system [16–18]. It is also the central core in sphingolipid metabolism, since it is a precursor of other bioactive sphingolipids [19]. DAG stimulates protein kinase C activity and adenylcyclase, which generates cAMP, a pivotal molecule in many biological processes [20]. DAG is also essential for propagation of the downstream signals required for NFkB activation. CER and DAG can be generated in cells in response to extracellular stimuli, such as cytokine, LPS [21] and LDL modified by oxidation or acetylation [22,23]. Besides activating the SM-CER pathway in cells, these modified LDLs can also carry ceramide itself [24].

The aim of the current study was to assess whether the products of the PLC-like activity generated during incubation of LDL(−) with cells, particularly CER and DAG, are involved in the inflammatory effect of LDL(−).

## 2. Results and Discussion

### 2.1. Lipid and Apolipoprotein Composition

The biochemical composition of LDL(−) has been studied when it is stored at 4 °C [25]. However, cultured cell studies to evaluate cytokine release are performed at 37 °C. When LDL(−) is incubated at 37 °C, some components of the lipoprotein could be modified. These modifications could provide or increase the inflammatory potential of LDL(−). Oxidative processes or phospholipolysis mediated by PLC-like enzymes could generate potentially pro-inflammatory lipids. In the current study, the biochemical composition of LDL(−) and native LDL (LDL(+)) was analyzed after 20 h at 37 °C and compared with samples kept at 4 °C. This was assayed in two conditions: untreated LDL subfractions or LDL subfractions pre-incubated for 2 h with HDL and then re-isolated. Table 1 shows the changes in lipoprotein composition of major lipids and apolipoproteins after 20 h of incubation at 37 °C and the effect of pre-incubation with HDL.

The major components of LDL(+) remained unchanged after incubation at 37 °C for 20 h, whereas LDL(−) increased its relative triglyceride (TG) and NEFA content and decreased the phospholipid (PL) content (Table 1). The relative increase in TG is not real, but should be explained by the PLC-like-mediated degradation of PL and the characteristics of the method for TG quantification. This is an enzyme-coupled method that uses triglyceride lipase for generating free glycerol and then glycerol kinase, glycerol-P oxidase and peroxidase. Since triglyceride lipase not only degrades TG, but also MAG and DAG, these molecules, which could be generated by the PLC-like activity, are detected as triglycerides.

Neither LDL(+) nor LDL(−) composition was modified in their major components by HDL pre-incubation, except for NEFA in LDL(−), which was partially transferred to HDL. These results are essentially the same as those previously reported [12] and support the exchange of NEFA between LDL(−) and HDL.

### 2.2. Phospholipolytic Activities

**Figure 1:**
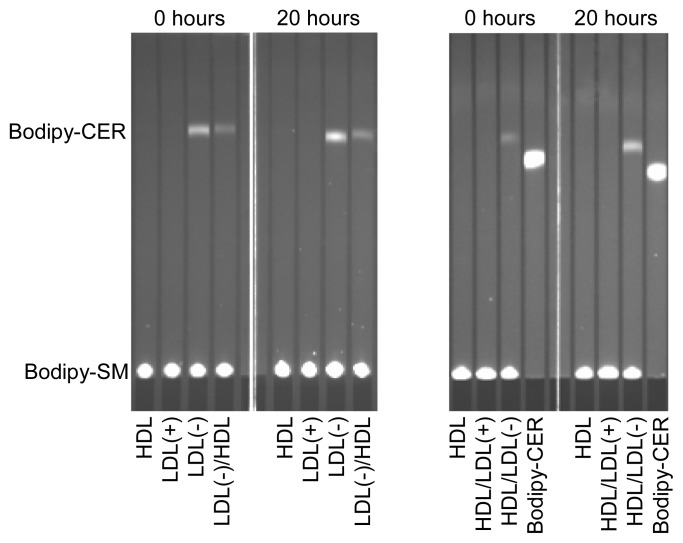
PLC-like activity (measured by the degradation of bodipy-labeled sphingomyelin [SM]) of LDL(+), LDL(−) and HDL before and after incubation at 37 °C for 20 h. Indicated samples were pre-incubated with the corresponding lipoprotein at 37 °C for 2 h. Bodipy-CER: bodipy-labeled ceramide. Bodipy-SM: bodipy-sphingomyelin. LDL(+)/HDL indicates LDL(+) that was pre-incubated with HDL for 2 h and re-isolated. Inversely, HDL/LDL(+) indicates HDL that was pre-incubated with LDL(+) for 2 h and re-isolated. The nomenclature of all samples follows the same pattern.

LDL(−) possesses increased PAF-AH and PLC-like activities compared to LDL(+) [6], which could be related with the generation of putative inflammatory lipids. We tested whether these enzymatic activities were modified after incubation at 37 °C. PAF-AH activity of the lipoproteins presented no significant difference after incubation at 37 °C for 20 h (data not shown). In contrast, the PLC-like activity was dramatically altered during incubation at 37 °C. The ability to degrade LPC and SM was evaluated by two methods: the Amplex Red method was used to quantify LPC degradation (Table 2), and the method combining bodipy-labeled SM and TLC was used to measure SM degradation (Figure 1). Both methods displayed similar results. In contrast to LDL(+), which showed no PLC-like activity, the PLC-like activity in LDL(−) increased after 20 h of incubation at 37 °C, and pre-incubation with HDL reduced this increase (Table 2 and Figure 1). This observation concurs with the decrease of PL observed in LDL(−) after 20 h of incubation at 37 °C (Table 1). On the other hand, and according to previous data, the PLC-like activity of LDL(−) was partially transferred to HDL [12]. This observation was corroborated by TLC using bodipy-SM as a substrate (Figure 1).

The cause of the increase in PLC-activity in LDL(−) incubated at 37 °C is unknown. Holopainen and colleagues suggested that apoB could have an intrinsic SMase activity, that is, a PLC activity specific for SM [26]. PLC-like activity is present in LDL(−) and absent in LDL(+), suggesting that PLC-like activity could be caused by conformational changes of apoB in LDL(−) that do not occur in LDL(+). However, this would not explain the surge of PLC-like activity in HDL. Its increase in HDL after incubation with LDL(−) suggests that some protein with this activity could be present in LDL(−). However, proteomic analysis of LDL(−) has not detected any protein with known PLC-like activity [27]. Further studies are needed to reveal the origin of the PLC-like activity associated with LDL(−).

On the other hand, we tested whether oxidative processes could occur during the incubation of samples at 37 °C. Table 2 (right column) shows that peroxide content, evaluated by the Auerbach method, did not differ among the samples in the conditions assayed. Cupper-oxidized LDL was used as positive control and presented a higher content in lipoperoxides (11.9 ± 3.3 μmol/g apoB, *n* = 6).

### 2.3. Products of PLC-Like Activity

**Figure 2:**
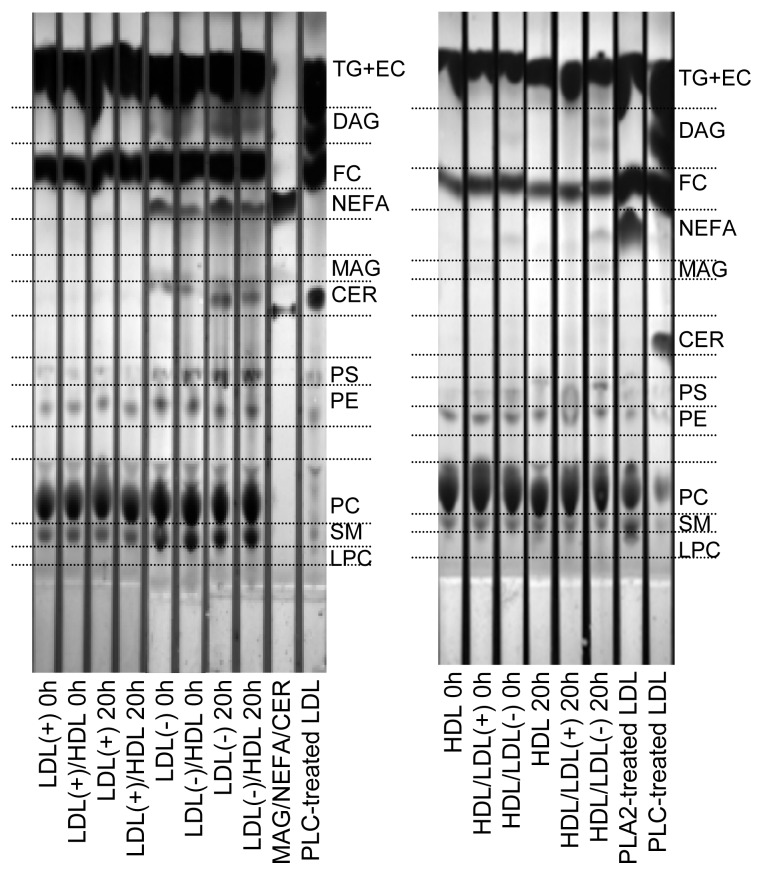
Representative TLC of lipid extracts of LDL(+), LDL(−) and HDL before and after incubation at 37 °C for 20 h. Indicated samples were pre-incubated with the corresponding lipoprotein at 37 °C for 2 h. LDL(+)/HDL indicates LDL(+) that was pre-incubated with HDL for 2 h and re-isolated. Inversely, HDL/LDL(+) indicates HDL that was pre-incubated with LDL(+) for 2 h and re-isolated. The nomenclature of all samples follows the same pattern. TG: triglyceride; EC: esterified cholesterol; DAG: diacylglycerol; FC: free cholesterol; MAG; monoacylglycerol; CER: ceramide; PS: phosphatidylserine; PE: phosphatidylcholine; PC: phosphatidylcholine; SM: sphingomyelin; LPC: lysophosphatidylcholine. A mixture of MAG/NEFA/CER or LDL modified with PLC or phospholipase A2 (PLA2) were used to identify the different spots.

The decrease of total PL observed in LDL(−) (Table 1) could be a consequence of the increase in PLC-like activity after 20 h at 37 °C (Table 2, Figure 1). Hence, we wanted to determine the putative parallel increase of the products resulting from the PLC-like activity, specifically CER, DAG and MAG. Figure 2 shows a representative TLC of the lipid extracts of lipoproteins. Compared to LDL(+), LDL(−) showed a higher content of NEFA, CER and DAG in basal conditions, and the amounts increased after incubation at 37 °C for 20 h. The content of MAG could not be clearly detected in LDLs. LDL(−) also showed a higher content of SM than LDL(+), in agreement with previous data [28]. Despite the increase of DAG and CER, the intensity of the spots corresponding to PC and SM, which are the origin of DAG and CER, respectively, did not decrease after 20 h of incubation at 37 °C. This observation indicates that only a minor amount of PC and SM is degraded by the PLC-like activity.

Pre-incubation of LDL(−) with HDL partially prevented the increase of NEFA, CER and DAG in LDL(−), probably due to their exchange with HDL, but also to the transfer of PLC-like activity. In contrast to LDL(−), neither LDL(+) nor HDL not pre-incubated with LDL(−) showed these products. HDL pre-incubated with LDL(−) increased its content in NEFA, DAG and MAG. Interestingly, the content of LPC in HDL decreased after incubation with LDL(−), in contrast to HDL or HDL pre-incubated with LDL(+). The decrease on LPC in HDL is probably the result of the gain in PLC-activity from LDL(−), which would efficiently hydrolyze LPC in HDL.

### 2.4. Effect of Incubation on LDL Aggregation

The lipoprotein basal aggregation level was evaluated by measuring absorbance at 450 nm (Table 3). We observed an increase of absorbance of LDL(−) after 20 h of incubation at 37 °C and a slight decrease in aggregation of LDL(−) after pre-incubation with HDL.

**Figure 3:**
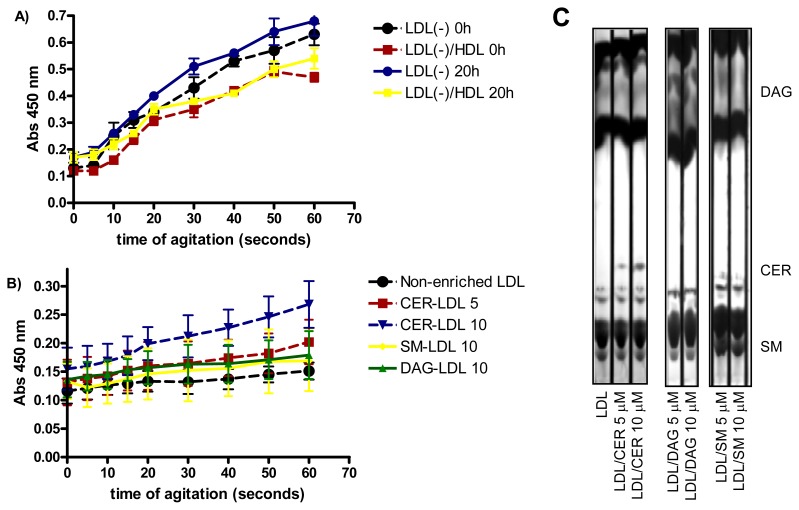
(**A**) LDL susceptibility to aggregation of LDL(−) before and after incubation at 37 °C for 20 h. LDL(−)-HDL indicates LDL(−) that were pre-incubated for 2 h with HDL (*n* = 3); (**B**) LDL susceptibility to aggregation of LDLs enriched *in vitro* with 5 and 10 μM ceramide (CER), 10 μM sphingomyelin (SM) and 10 μM diacylglycerol (DAG) (*n* = 3); (**C**) TLC showing the enrichment of LDL with 5 and 10 μM ceramide (CER), diacylglycerol (DAG) and sphingomyelin (SM) (*n* = 3).

LDL susceptibility to aggregation *in vitro* was also monitored after intense agitation by vortex. As previously reported [29], LDL(−) was more susceptible to aggregation than LDL(+) (data not shown), Incubation of LDL(−) at 37 °C for 20 h renders the particle more prone to aggregation than at 4 °C (Figure 3A). The high susceptibility to aggregation of LDL(−) diminished when it was pre-incubated with HDL, probably because HDL decreases, in parallel with PLC-like activity, the content of CER and DAG in LDL(−). These lipids favor LDL aggregation [15,16]. Accordingly, we assessed whether the enrichment of LDL in CER, DAG and SM (Figure 3C) increased susceptibility to aggregation. Figure 3B shows that LDL enriched in CER was more prone to aggregation than non-enriched LDL, in a dose-dependent manner. In contrast, LDL enriched in DAG was not more susceptible to aggregation than non-enriched LDL. These data support a relationship between CER content and aggregability of LDL.

### 2.5. Relationship Between Aggregation of LDL(−) and Cytokine Release

**Figure 4:**
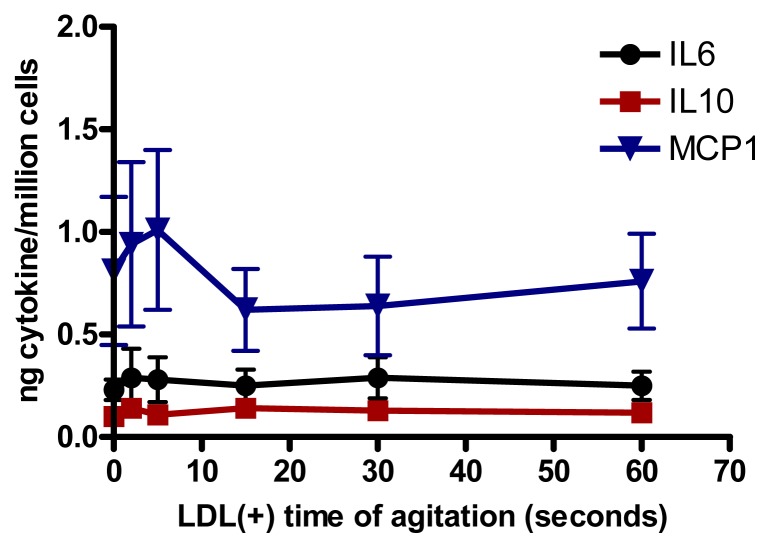
IL6, IL10 and MCP1 release induced in monocytes by LDL (150 mg apolipoprotein B[apoB]/L) aggregated by intense agitation (*n* = 5).

We assessed whether the increased aggregation level in LDL(−) observed at 37 °C could be involved in cytokine release induced in monocytes. Native LDL was aggregated *in vitro* by intense agitation. The aggregation level (absorbance at 450 nm) of LDL(−) after 20 h of incubation at 37 °C is achieved with native LDL vortexed for 40–60 s. LDLs with different aggregation levels were incubated with monocytes. We observed that LDL was able to induce the release of IL6, IL10 and MCP1 in cultured monocytes at any time of agitation (Figure 4). These results show that the cytokine release promoted by LDL(−) was not caused by its physical aggregation, but by other chemical modifications.

### 2.6. Involvement of PLC-Like Activity Products in Cytokine Release

We aimed to assess whether the major products of PLC-like activity formed in LDL(−) could play a role in the cytokine release promoted by LDL(−) on monocytes. Table 4 shows the release of IL6 induced by modified LDLs in monocytes. PLC-treated LDL (50 and 100 U/L), which leads to an increase in CER and DAG content in LDL, induced a dose-dependent increase of IL6. However, PLC-induced lipolysis did not allow us to distinguish between the involvement of either CER or DAG. We therefore tested LDLs selectively enriched with CER, DAG or SM. Enrichment of LDL in DAG or SM did not significantly induce cytokine release compared to non-enriched LDL (Table 4). In contrast, the supplementation of LDL with CER stimulated IL6 release compared to non-enriched LDL.

**Figure 5:**
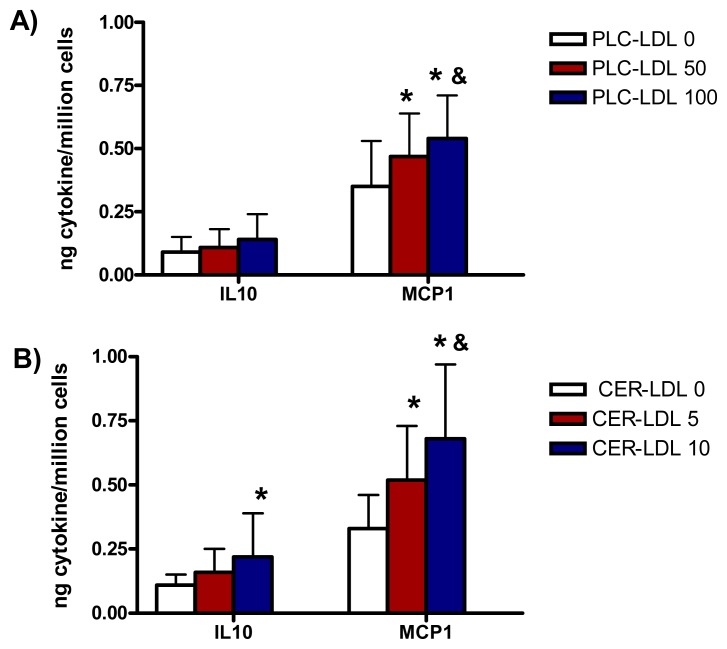
IL10 and MCP1 release induced in monocytes by LDLs (150 mg apoB/L) modified with PLC (PLC-LDL, **A**) or enriched with CER (CER-LDL, **B**) (*n* = 5) *p < 0.05 *vs*. PLC-LDL 0 or CER-LDL 0. ^&^*p* < 0.05 *vs*. PLC-LDL 50 or CER-LDL 5.

The incubation of monocytes with PLC-LDL and CER-LDL promoted a release of IL10 and MCP1 similar to the release of IL6 (Figure 5).

## 3. Experimental Section

### 3.1. Lipoprotein Isolation and Separation of LDL Subfractions

Plasma samples from healthy normolipemic subjects (total cholesterol < 5.2 mmol/L, triglyceride < 1 mmol/L) were obtained in EDTA-containing Vacutainer tubes. LDL (density range 1.019–1.050 g/mL) and HDL (1.100–1.210 g/mL) were isolated by sequential flotation ultracentrifugation at 4 °C. Total LDL was subfractionated in LDL(+) and LDL(−) by preparative anion-exchange chromatography in an ÄKTA-FPLC system (GE Healthcare, Chalfont Sant Giles, UK) [29]. In all experiments, the proportion of LDL(−) ranged from 4% to 6% of total LDL, and the major characteristics of both LDL subfractions were similar to those previously reported [25] (data not shown). Both subfractions were concentrated by centrifugation with Amicon microconcentrators. All the analyses with these subfractions were performed within 3 days of isolation.

### 3.2. LDL Incubation with HDL

LDL(+) and LDL(−) (0.5 g/L apoB) were incubated with HDL (0.5 g/L apoAI) at 37 °C for 2 h in phosphate saline buffer (PBS) and shaken gently in the presence of 20 μM butylated hydroxytoluene (BHT) to avoid oxidation. After incubation, LDL and HDL were re-isolated according to their density. Half of each sample was kept at 4 °C, and the other half was incubated at 37 °C for 20 h to reproduce the conditions of cytokine release experiments with cells. Afterwards, samples were kept at 4 °C until the physicochemical characterization.

### 3.3. LDL Modification

To selectively enrich LDL with PLC-like activity products, liposomes containing these different compounds were used as donor and native LDL was used as acceptor, as described [30]. To form the liposomes, total lipids were extracted from native LDL (1 mg apoB), according to the Bligh and Dyer method, and the compounds were added to lipids from the chloroform phase. *N*-acetyl-d-sphingosine (ceramide, CER), 1,2-diacyl-sn-glycero-3-phosphocholine (diacylglycerol, DAG) and sphingomyelin (SM) were added at 5 and 10 μM. All compounds were from Sigma. Afterwards, the lipid extract was dried under a nitrogen stream, resuspended in 200 μL of a KBr solution (density 1.019 g/mL) and sonicated in a water bath until the mixture was translucent, which indicates that liposomes are formed. The acceptor (LDL at 1 mg apoB) was then incubated with the donor vesicle (liposomes) at 37 °C for 45 min in the presence of 20 μM BHT. After the incubation, the suspension was overlaid with KBr solution (density 1.019 g/mL), and liposomes and LDL were re-isolated by ultracentrifugation. Liposomes, which are less dense than 1.019 g/mL, were recovered floating at the surface, while enriched-LDL (CER-LDL, DAG-LDL, SM-LDL) was recovered in the lower phase. Non-enriched LDL was processed in parallel and incubated with non-enriched liposomes. Lipid extracts of the samples were separated by thin layer chromatography (TLC), to assess the increase of DAG, CER and SM.

LDL(+) (0.5 g/L) was modified with PLC (Sigma, Madrid, Spain) by incubation with the enzyme at 50 and 100 U/L for 1 h at 37 °C. The reaction buffer was 5 mM HEPES, 2 mM CaCl_2_, 5 mM MgCl_2_, 140 mM NaCl at pH 7.4. The reaction was stopped with 10 mM EDTA. LDL(+) was aggregated *in vitro* by vortexing for increasing times.

### 3.4. Lipid and Apoprotein Composition

Cholesterol, triglyceride, apoB, apoAI (Roche Diagnostic, Basel, Switzerland), phospholipid and NEFA (Wako Chemicals, Richmond, VA, USA) content of the samples were determined in a Hitachi 917 autoanalyzer. The results were expressed as the percentage of lipoprotein mass and, in the case of NEFA, as mol NEFA/mol apoB for LDL and mol NEFA/mol apoAI for HDL.

Other minor lipid components, including CER, DAG and MAG, were evaluated by extracting lipids from LDLs (250 μg apoB), according to the Bligh and Dyer method. Lipid extracts were resuspended in 20 μL chloroform and applied to the TLC plate. The silica gel plates were developed using three sequential mobile phases, as follows. Phase 1: chloroform/methanol/water (*v*/*v*/*v* 65:40:5) to 5 cm. Phase 2: toluene/diethilether/ethanol (*v*/*v*/*v* 60:40:3) to 13 cm. Phase 3: heptane to 17 cm. Lipids were stained by dipping the plates in a solution containing 5% phosphomolibdic and 5% sulfuric acid in ethanol for 1 min, and then dried at 100 °C for 10 min.

### 3.5. Aggregation Level and Oxidation Tests

Aggregation was monitored by measuring absorbance of LDL and HDL at 450 nm (0.5 g/L apoB or apoAI for basal aggregation and 0.2 g/L for susceptibility to aggregation). Lipoproteins were vortexed at increasing times up to 1 min [12].

The peroxide content of lipoproteins was measured following the Auerbach assay, using 20 μg of apoB for LDL or apoAI for HDL [31].

### 3.6. Enzymatic Activity Measurements

PAF-AH activity of the samples at 0.2 g/L apoB (LDL) and apoAI (HDL) was evaluated by a commercial colorimetric assay based on degradation of 2-thio-PAF (Cayman Chemical, Michigan, MI, USA) [5]. PLC-like activity of LDL(+), LDL(−), HDL and apoAI was evaluated in the different incubation conditions [6]. Briefly, we used a commercial fluorimetric assay based on enzyme-coupled reactions and final detection of fluorescent Amplex red. Sphingomyelin (SM) and lysophosphatidylcholine (LPC) were used as substrates, and 30 μg of lipoproteins (apoB in LDLor apoAI in HDL) were assayed. Fluorescence was monitored for 3 h and activity was calculated from the maximum slope.

To corroborate the SMase activity and avoid possible interferences of the Amplex red method, the degradation of SM labeled with the fluorescent probe bodipy (bodipy-SM) (Molecular Probes, Leiden, The Netherlands) was also measured. As described, samples (50 μg) were incubated with bodipy-SM for 3 h at 37 °C, followed by lipid extraction and separation by TLC [6].

### 3.7. Cytokine Release Experiments in Monocytes

Human monocytes were isolated according to their density from the blood of normal volunteers who gave their written informed consent [13]. Monocytes were cultured in 12-well plates (10^6^ cells/well) for 1 day and incubated in the experiments, with LDL previously filtered in sterility, at 150 mg apoB/L for 20 h, in the case of LDL. After incubation, the supernatant was collected, and the concentrations of IL6, IL10 and MCP1 were quantified by ELISA (Bender Medsystems, Burlingame, CA, USA) [13].

### 3.8. Statistical Analysis

Results are expressed as mean ± SD. A Sigma Stat 2.0 statistical package was used. Differences between groups were tested with Wilcoxon’s *T*-test (for paired data).

## 4. Discussion

Our results show a relationship between the content of CER in LDL(−) and the release of cytokines in monocytes. In contrast, in our experimental conditions, DAG-LDL did not promote a significant increase of IL6 release compared to native LDL. LDL(−) pre-incubated with HDL decreased its PLC-activity and CER content. This effect could explain the described counteracting effect of HDL on LDL(−) inflammatory action [12]. It is known that CER acts as a second messenger in signal transduction pathways in cells, including biosynthesis of cytokines [18,32]. A relationship between the content of CER in LDL and the development of inflammatory diseases, including atherosclerosis, has been described [24,33]. Retained lesional LDL also accumulates CER [15,24]. The involvement of CER in cytokine release promoted by LDL(−) could be related to the recent observation of CD14 as the main receptor of LDL(−) in monocytes [34]. The binding of LDL(−) to CD14 could be mediated by CER, as it has been reported that CD14 binds CER [35]. This binding activates the TLR4 assembly and cytokine induction in monocytes. Interestingly, CD14 also binds NEFA [36]. The observation that both CER and NEFA increase their content in LDL(−) after 20 h of incubation at 37 °C supports the role of CD14 in the activation of the inflammatory response mediated by LDL(−) in monocytes.

Besides promoting hydrolysis of its own phospholipids, the high PLC-like activity of LDL(−) could induce a degradation of phospholipids in the membrane of monocytes. This could promote an increase of the intracellular CER content and its derivates. This effect was also reported for oxLDL [37]. In addition, LDL(−) could activate cell SMase activity, since it induces Fas expression in monocytes [38], an action known to promote SM breakdown and CER production [39].

Our data show that CER-LDL released more cytokines than native LDL, but not as much as the release induced by LDL(−). CER-LDL did not reproduce all the characteristics of LDL(−), suggesting the involvement of other compounds in the inflammatory effect of LDL(−). Besides NEFA, CER metabolites, such as sphingosine and sphingosine-1-phosphate, could also be involved [40–43]. It has been proposed that HDL prevents NF-kB activation and synthesis of inflammatory proteins by decreasing sphingosine-1-phosphate [44]. The decrease in CER content in LDL(−) when incubated with HDL could also cause a decrease in these CER metabolites.

## 5. Conclusions

The current work provides new insights into the properties of LDL(−) involved in the cytokine release promoted in monocytes and the mechanisms by which HDL inhibits this action. We report that the increased CER content in LDL(−) that comes from its own PLC-like activity is related to its susceptibility to aggregation and its inflammatory effect on monocytes. These properties of LDL(−) are inhibited by HDL. More studies are necessary to ascertain the involvement of CER and PLC-like activity in the specific mechanisms activated by LDL(−) in monocytes.

## Figures and Tables

**Table 1:** Changes of lipoprotein composition after incubation of low-density lipoprotein (LDL)(+), LDL(−) and high-density lipoprotein (HDL) at 37 °C for 20 h. Indicated samples were pre-incubated with the corresponding lipoprotein at 37 °C for 2 h.

	Pre-incubation	Time of incubation	% CT	% TG	% PL	% apoB or apoAI	NEFA (mol/mol apo)
LDL(+)	-	0 h	42.3 ± 2.4	7.0 ± 1.2	28.8 ± 3.4	21.9 ± 2.5	9.7 ± 4.7
	HDL	0 h	42.6 ± 1.7	6.8 ± 1.2	29.1 ± 2.7	21.4 ± 1.9	8.2 ± 5.2
	-	20 h	42.3 ± 1.7	6.8 ± 1.1	28.1 ± 2.9	22.8 ± 2.7	11.7 ± 5.6
	HDL	20 h	42.2 ± 1.8	7.2 ± 1.4	27.8 ± 3.2	22.8 ± 2.3	9.6 ± 4.1
LDL(−)	-	0 h	42.7 ± 2.3	10.6 ± 0.9 #	26.4 ± 2.4 #	20.3 ± 2.8 #	32.2 ± 7.5 #
	HDL	0 h	43.3 ± 2.2	10.6 ± 1.3	26.3 ± 2.2	19.9 ± 1.6	19.7 ± 3.4
	-	20 h	42.6 ± 1.6	15.1 ± 1.5 #*	22.0 ± 3.4 #*	20.3 ± 2.7 #	57.0 ± 17.4 #*
	HDL	20 h	42.4 ± 1.3	13.8 ± 1.3 *	23.4 ± 2.4 *	20.4 ± 2.7	42.3 ± 10.2
HDL	-	0 h	16.8 ± 2.7	4.1 ± 1.0	32.5 ± 4.6	46.0 ± 7.4	0.1 ± 0.1
	LDL(+)	0 h	18.0 ± 2.3	4.1 ± 0.8	33.3 ± 3.8	44.6 ± 6.0	0.2 ± 0.1
	LDL(−)	0 h	20.1 ± 2.6 &	5.4 ± 1.0 &	31.8 ± 3.6	42.7 ± 5.6	0.8 ± 0.4 &
	-	20 h	16.3 ± 3.4	3.7 ± 1.2	31.3 ± 6.2	47.9 ± 10.5	0.2 ± 0.1
	LDL(+)	20 h	17.8 ± 2.9	4.6 ± 1.2	31.7 ± 4.6	45.9 ± 7.9	0.3 ± 0.2
	LDL(−)	20 h	20.0 ± 5.5 &	9.7 ± 4.5 *&	27.1 ± 4.7 *&	43.2 ± 12.0	1.6 ± 0.9 *&

CT: total cholesterol; TG: triglycerides; PL: phospholipids; apo: apolipoprotein; NEFA: non-esterified fatty acids. Data are the mean ± SD (*n* = 6) expressed as % of total mass, except NEFA that are expressed as mol NEFA/mol apo.

# indicates *p* < 0.05 LDL(−) *vs*. LDL(+);

* indicates *p* < 0.05 20 h at 37 °C *vs*. 0 h;

& indicates *p* < 0.05 untreated LDL or HDL *vs*. pre-incubated LDL or HDL.

**Table 2:** Phospholipase C(PLC)-like activity (measured by the Amplex Red method) and peroxide content of LDL(+), LDL(−) and HDL before and after incubation at 37 °C for 20 h. Indicated samples were pre-incubated with the corresponding lipoprotein at 37 °C for 2 h.

	Pre-incubation	Time of incubation	PLC-like activity (mU equivalents/mg apo)	μmol peroxides/g apo
LDL(+)	-	0 h	4.5 ± 8.7	4.88 ± 0.63
	HDL	0 h	0.6 ± 0.8	3.77 ± 1.16
	-	20 h	6.0 ± 6.1	4.69 ± 1.18
	HDL	20 h	5.1 ± 3.8	3.70 ± 1.28
LDL(−)	-	0 h	69.3 ± 15.6 #	4.66 ± 1.01
	HDL	0 h	48.7 ± 9.8 &	3.85 ± 0.20
	-	20 h	201.5 ± 35.5 #*	3.92 ± 0.51
	HDL	20 h	134.9 ± 38.4 *&	4.47 ± 0.67
HDL	-	0 h	0.2 ± 0.2	6.34 ± 2.47
	LDL(+)	0 h	0.3 ± 0.3	6.44 ± 2.55
	LDL(−)	0 h	72.4 ± 16.9 &	6.54 ± 2.30
	-	20 h	0.2 ± 0.1	5.24 ± 0.81
	LDL(+)	20 h	0.7 ± 0.7	5.14 ± 1.37
	LDL(−)	20 h	165.5 ± 27.9 *&	5.08 ± 1.22

Data are the mean ± SD (*n* = 6).

# indicates *p* < 0.05 LDL(−) *vs*. LDL(+),

* indicates *p* < 0.05 20 h at 37 °C *vs*. 0 h,

& indicates *p* < 0.05 LDL pre-incubated with HDL *vs*. non-pre-incubated.

**Table 3:** Absorbance at 450 nm of LDL(+), LDL(−) and HDL before and after incubation at 37 °C for 20 h. Indicated samples were pre-incubated with the corresponding lipoprotein at 37 °C for 2 h.

	Pre-incubation	Time of incubation	Absorbance 450 nm (abs units)
LDL(+)	-	0 h	0.092 ± 0.005
	HDL	0 h	0.088 ± 0.009
	-	20 h	0.101 ± 0.008
	HDL	20 h	0.101 ± 0.006
LDL(−)	-	0 h	0.101 ± 0.015 #
	HDL	0 h	0.090 ± 0.006 &
	-	20 h	0.152 ± 0.017 #*
	HDL	20 h	0.130 ± 0.028 *#&
HDL	-	0 h	0.046 ± 0.007
	LDL(+)	0 h	0.046 ± 0.005
	LDL(−)	0 h	0.053 ± 0.008 &
	-	20 h	0.047 ± 0.008
	LDL(+)	20 h	0.047 ± 0.010
	LDL(−)	20 h	0.049 ± 0.008

Data are the mean ± SD (*n* = 6).

# indicates *p* < 0.05 LDL(−) *vs*. LDL(+),

* indicates *p* < 0.05 20 h at 37 °C *vs*. 0 h,

& indicates *p* < 0.05 LDL pre-incubated with HDL *vs*. non-pre-incubated.

**Table 4:** IL6 release induced by LDLs (150 mg apoB/L) in monocytes after 20 h of incubation (*n* = 5).

	IL6 release (ng/million cells)
LDL(+)	0.30 ± 0.06
LDL(−)	0.64 ± 0.09 *
Non-enriched LDL	0.33 ± 0.09
PLC-LDL (50 U/L)	0.38 ± 0.09
PLC-LDL (100 U/L)	0.56 ± 0.12 *
CER-LDL (5 μM)	0.45 ± 0.12 *
CER-LDL (10 μM)	0.55 ± 0.15 *
DAG-LDL (5 μM)	0.32 ± 0.08
DAG-LDL (10 μM)	0.35 ± 0.12
SM-LDL (5 μM)	0.33 ± 0.04
SM-LDL (10 μM)	0.36 ± 0.10

* *p* < 0.05 *vs*. LDL(+) or non-enriched LDL.

## References

[1] Navab M., Berliner J.A., Watson A.D., Hama S.Y., Territo M.C., Lusis A.J., Shih D.M., van Lenten B.J., Frank J.S., Demer L.L. (1996). The Yin and Yang of oxidation in the development of the fatty streak. A review based on the 1994 George Lyman Duff Memorial Lecture. Arterioscler. Thromb. Vasc. Biol.

[2] Sanchez-Quesada J.L., Benitez S., Ordonez-Llanos J. (2004). Electronegative low-density lipoprotein. Curr. Opin. Lipidol.

[3] Benitez S., Camacho M., Arcelus R., Vila L., Bancells C., Ordonez-Llanos J., Sanchez-Quesada J.L. (2004). Increased lysophosphatidylcholine and non-esterified fatty acid content in LDL induces chemokine release in endothelial cells. Relationship with electronegative LDL. Atherosclerosis.

[4] Benitez S., Sanchez-Quesada J.L., Lucero L., Arcelus R., Ribas V., Jorba O., Castellvi A., Alonso E., Blanco-Vaca F., Ordonez-Llanos J. (2002). Changes in low-density lipoprotein electronegativity and oxidizability after aerobic exercise are related to the increase in associated non-esterified fatty acids. Atherosclerosis.

[5] Benitez S., Sanchez-Quesada J.L., Ribas V., Jorba O., Blanco-Vaca F., Gonzalez-Sastre F., Ordonez-Llanos J. (2003). Platelet-activating factor acetylhydrolase is mainly associated with electronegative low-density lipoprotein subfraction. Circulation.

[6] Bancells C., Benitez S., Villegas S., Jorba O., Ordonez-Llanos J., Sanchez-Quesada J.L. (2008). Novel phospholipolytic activities associated with electronegative low-density lipoprotein are involved in increased self-aggregation. Biochemistry.

[7] Sanchez-Quesada J.L., Villegas S., Ordonez-Llanos J. (2012). Electronegative LDL: A link between apoB misfolding, lipoprotein aggregation and proteoglycan binding. Curr. Opin. Lipidol.

[8] Bancells C., Villegas S., Blanco F.J., Benitez S., Gallego I., Beloki L., Perez-Cuellar M., Ordonez-Llanos J., Sanchez-Quesada J.L. (2010). Aggregated electronegative low density lipoprotein in human plasma shows a high tendency toward phospholipolysis and particle fusion. J. Biol. Chem.

[9] Bancells C., Benitez S., Ordonez-Llanos J., Oorni K., Kovanen P.T., Milne R.W., Sanchez-Quesada J.L. (2011). Immunochemical analysis of the electronegative LDL subfraction shows that abnormal *N*-terminal apolipoprotein B conformation is involved in increased binding to proteoglycans. J. Biol. Chem.

[10] Bancells C., Benitez S., Jauhiainen M., Ordonez-Llanos J., Kovanen P.T., Villegas S., Sanchez-Quesada J.L., Oorni K. (2009). High binding affinity of electronegative LDL to human aortic proteoglycans depends on its aggregation level. J. Lipid Res.

[11] Benitez S., Camacho M., Bancells C., Vila L., Sanchez-Quesada J.L., Ordonez-Llanos J. (2006). Wide proinflammatory effect of electronegative low-density lipoprotein on human endothelial cells assayed by a protein array. Biochim. Biophys. Acta.

[12] Bancells C., Sanchez-Quesada J.L., Birkelund R., Ordonez-Llanos J., Benitez S. (2010). HDL and electronegative LDL exchange anti- and pro-inflammatory properties. J. Lipid Res.

[13] Benitez S., Bancells C., Ordonez-Llanos J., Sanchez-Quesada J.L. (2007). Pro-inflammatory action of LDL(−) on mononuclear cells is counteracted by increased IL10 production. Biochim. Biophys. Acta.

[14] Oorni K., Pentikainen M.O., Ala-Korpela M., Kovanen P.T. (2000). Aggregation, fusion, and vesicle formation of modified low density lipoprotein particles: Molecular mechanisms and effects on matrix interactions. J. Lipid Res.

[15] Schissel S.L., Tweedie-Hardman J., Rapp J.H., Graham G., Williams K.J., Tabas I. (1996). Rabbit aorta and human atherosclerotic lesions hydrolyze the sphingomyelin of retained low-density lipoprotein. Proposed role for arterial-wall sphingomyelinase in subendothelial retention and aggregation of atherogenic lipoproteins. J. Clin. Invest.

[16] Mathias S., Pena L.A., Kolesnick R.N. (1998). Signal transduction of stress via ceramide. Biochem. J.

[17] Laulederkind S.J., Bielawska A., Raghow R., Hannun Y.A., Ballou L.R. (1995). Ceramide induces interleukin 6 gene expression in human fibroblasts. J. Exp. Med.

[18] Ballou L.R., Laulederkind S.J., Rosloniec E.F., Raghow R. (1996). Ceramide signalling and the immune response. Biochim. Biophys. Acta.

[19] Arana L., Gangoiti P., Ouro A., Trueba M., Gomez-Munoz A. (2010). Ceramide and ceramide 1-phosphate in health and disease. Lipids Health Dis.

[20] Hajjar D.P., Pomerantz K.B. (1992). Signal transduction in atherosclerosis: Integration of cytokines and the eicosanoid network. FASEB J.

[21] Sweet M.J., Hume D.A. (1996). Endotoxin signal transduction in macrophages. J. Leukoc. Biol.

[22] Kinscherf R., Claus R., Deigner H.P., Nauen O., Gehrke C., Hermetter A., Russwurm S., Daniel V., Hack V., Metz J. (1997). Modified low density lipoprotein delivers substrate for ceramide formation and stimulates the sphingomyelin-ceramide pathway in human macrophages. FEBS Lett.

[23] Grandl M., Bared S.M., Liebisch G., Werner T., Barlage S., Schmitz G. (2006). E-LDL and Ox-LDL differentially regulate ceramide and cholesterol raft microdomains in human macrophages. Cytometry A.

[24] Lightle S., Tosheva R., Lee A., Queen-Baker J., Boyanovsky B., Shedlofsky S., Nikolova-Karakashian M. (2003). Elevation of ceramide in serum lipoproteins during acute phase response in humans and mice: Role of serine-palmitoyl transferase. Arch. Biochem. Biophys.

[25] De Castellarnau C., Sanchez-Quesada J.L., Benitez S., Rosa R., Caveda L., Vila L., Ordonez-Llanos J. (2000). Electronegative LDL from normolipemic subjects induces IL-8 and monocyte chemotactic protein secretion by human endothelial cells. Arterioscler. Thromb. Vasc. Biol.

[26] Holopainen J.M., Medina O.P., Metso A.J., Kinnunen P.K. (2000). Sphingomyelinase activity associated with human plasma low density lipoprotein. J. Biol. Chem.

[27] Bancells C., Canals F., Benítez S., Colomé N., Julve J., Ordóñez-Llanos J., Sanchez-Quesada J. (2010). Proteomic analysis of electronegative low density lipoprotein. J. Lipid Res.

[28] Sevanian A., Bittolo-Bon G., Cazzolato G., Hodis H., Hwang J., Zamburlini A., Maiorino M., Ursini F. (1997). LDL-is a lipid hydroperoxide-enriched circulating lipoprotein. J. Lipid Res.

[29] Sanchez-Quesada J.L., Camacho M., Anton R., Benitez S., Vila L., Ordonez-Llanos J. (2003). Electronegative LDL of FH subjects: Chemical characterization and induction of chemokine release from human endothelial cells. Atherosclerosis.

[30] Boyanovsky B., Karakashian A., King K., Giltiay N., Nikolova-Karakashian M. (2003). Uptake and metabolism of low density lipoproteins with elevated ceramide content by human microvascular endothelial cells: Implications for the regulation of apoptosis. J. Biol. Chem.

[31] Auerbach B.J., Kiely J.S., Cornicelli J.A. (1992). A spectrophotometric microtiter-based assay for the detection of hydroperoxy derivatives of linoleic acid. Anal. Biochem.

[32] Serhan C.N., Haeggstrom J.Z., Leslie C.C. (1996). Lipid mediator networks in cell signaling: Update and impact of cytokines. FASEB J.

[33] Chatterjee S. (1998). Sphingolipids in atherosclerosis and vascular biology. Arterioscler. Thromb. Vasc. Biol.

[34] Estruch M., Sanchez-Quesada J.L., Bancells C., Beloki L., Ordonez-Llanos J., Benitez S Involvement of CD14 and TLR4 in the binding of electronegative LDL and consequent cytokine release in monocytes.

[35] Pfeiffer A., Bottcher A., Orso E., Kapinsky M., Nagy P., Bodnar A., Spreitzer I., Liebisch G., Drobnik W., Gempel K. (2001). Lipopolysaccharide and ceramide docking to CD14 provokes ligand-specific receptor clustering in rafts. Eur. J. Immunol.

[36] Suganami T., Tanimoto-Koyama K., Nishida J., Itoh M., Yuan X., Mizuarai S., Kotani H., Yamaoka S., Miyake K., Aoe S. (2007). Role of the Toll-like receptor 4/NF-kappaB pathway in saturated fatty acid-induced inflammatory changes in the interaction between adipocytes and macrophages. Arterioscler. Thromb. Vasc. Biol.

[37] Auge N., Nikolova-Karakashian M., Carpentier S., Parthasarathy S., Negre-Salvayre A., Salvayre R., Merrill A.H., Levade T. (1999). Role of sphingosine 1-phosphate in the mitogenesis induced by oxidized low density lipoprotein in smooth muscle cells via activation of sphingomyelinase, ceramidase and sphingosine kinase. J. Biol. Chem..

[38] Bancells C., Sánchez-Quesada J.L., Birkelund R., Ordóñez-Llanos J., Benítez S. (2010). Electronegative LDL induces Fas and modifies gene expression in mononuclear cells. Front. Biosci.

[39] Cifone M.G., de Maria R., Roncaioli P., Rippo M.R., Azuma M., Lanier L.L., Santoni A., Testi R. (1994). Apoptotic signaling through CD95 (Fas/Apo-1) activates an acidic sphingomyelinase. J. Exp. Med.

[40] Spiegel S., Milstien S. (2003). Sphingosine-1-phosphate: An enigmatic signalling lipid. Nat. Rev. Mol. Cell. Biol.

[41] Chandru H., Boggaram V. (2007). The role of sphingosine 1-phosphate in the TNF-alpha induction of IL-8 gene expression in lung epithelial cells. Gene.

[42] Lai W.Q., Irwan A.W., Goh H.H., Howe H.S., Yu D.T., Valle-Onate R., McInnes I.B., Melendez A.J., Leung B.P. (2008). Anti-inflammatory effects of sphingosine kinase modulation in inflammatory arthritis. J. Immunol.

[43] Mao C., Obeid L.M. (2008). Ceramidases: Regulators of cellular responses mediated by ceramide, sphingosine, and sphingosine-1-phosphate. Biochim. Biophys. Acta.

[44] Barter P.J., Nicholls S., Rye K.A., Anantharamaiah G.M., Navab M., Fogelman A.M. (2004). Antiinflammatory properties of HDL. Circ. Res.

